# A Multi-National Questionnaire-Based Analysis of Dental Students’ Knowledge of the Management of Deep Caries and the Exposed Pulp

**DOI:** 10.1016/j.identj.2025.100844

**Published:** 2025-06-06

**Authors:** Venkateshbabu Nagendrababu, Arindam Dutta, Ana Arias, Aleksandar Jakovljevic, Ahmed Hieawy, Frank C. Setzer, Jugoslav Ilic, Milos Beloica, Meric Karapinar Kazandag, Nandini Suresh, Paul V. Abbott, Raghavendra M. Shetty, Srinivasan Narasimhan, Victoria SH Yu, Vellore Kannan Gopinath, Ya Shen, Henry F. Duncan

**Affiliations:** a Department of Restorative Dentistry, College of Dental Medicine, University of Sharjah, Sharjah, UAE; b School of Dentistry, College of Biomedical and Lifesciences, Cardiff University, Cardiff, United Kingdom; c Department of Conservative and Prosthetic Dentistry, School of Dentistry, Complutense University, Madrid, Spain; d Department of Pathophysiology, School of Dental Medicine, University of Belgrade, Belgrade, Serbia; e Department of Oral Biological & Medical Sciences, Faculty of Dentistry, University of British Columbia, Vancouver, Canada; f Department of Endodontics, School of Dental Medicine, University of Pennsylvania, Philadelphia, Pennsylvania, USA; g Department of Restorative Odontology and Endodontology, School of Dental Medicine, University of Belgrade, Belgrade, Serbia; h Department of Pediatric and Preventive Dentistry, School of Dental Medicine, University of Belgrade; i Faculty of Dentistry, Department of Endodontics, Yeditepe University, Ataşehir - İstanbul, Turkley; j Louisiana State University Health Sciences Center, School of Dentistry, Department of Endodontics, New Orleans, Louisiana, USA; k Meenakshi Ammal Dental College and Hospital, Meenakshi Academy of Higher Education and Research (MAHER), Tamil Nadu, India; l UWA Dental School, The University of Western Australia, Perth, Australia; m Department of Clinical Sciences, College of Dentistry, Ajman University, Ajman, UAE; n Center of Medical and Bio‑Allied Health Sciences Research, Ajman University, Ajman, UAE; o Department of Pediatric and Preventive Dentistry, International Adjunct Faculty, Sharad Pawar Dental College and Hospital, Datta Meghe Institute of Higher Education and Research (Declared as Deemed-to-be University), Wardha, Maharashtra, India; p Hamad Dental Center, Hamad Medical Corporation, Doha, Qatar; q Faculty of Dentistry, National University of Singapore (NUS), Singapore, Singapore; r College of Dental Medicine, Department of Orthodontics, Pediatric and Community Dentistry, University of Sharjah, Sharjah, UAE; s Division of Restorative Dentistry, Dublin Dental University Hospital, Trinity College Dublin, Dublin, Ireland

**Keywords:** Deep caries, Exposed pulp, Pulp capping, Survey, Root canal treatment, Vital pulp treatment

## Abstract

**Introduction and aims:**

To evaluate knowledge regarding the management of deep carious lesions and exposed pulps among undergraduate and postgraduate endodontic students from ten dental institutions across ten countries, and the impact of operator (material, antibiotic prescription) and patient-related (age, symptoms) factors on their treatment protocols.

**Methods:**

An online questionnaire was distributed to evaluate student knowledge of the management of deep caries and exposed pulp related to four clinical scenarios. Simple descriptive statistics were used to describe the data and McNemar tests were employed to identify significant differences between the scenarios. The P-value was set at 5%.

**Results:**

A total of 435 undergraduates and 139 postgraduates from ten dental schools participated in this survey. The final survey included 401 responses from undergraduates and 127 from postgraduates for statistical analysis. When symptoms were present, the majority of undergraduate and postgraduate students preferred non-selective (complete) caries removal over selective (partial) caries removal in young patients. The majority of postgraduates preferred partial pulpotomy in younger patients and pulpectomy and root canal treatment (RCT) in older patients. The majority of undergraduates preferred pulpectomy and RCT in both young/old patients when symptoms were present. The majority of undergraduates and postgraduates opted for mineral trioxide aggregate and Biodentine, respectively, when treating the exposed pulp. Systemic antibiotics were not recommended by both undergraduates and postgraduates, regardless of the patient's age and symptoms.

**Conclusion:**

Among the scenarios surveyed, the majority of undergraduates and postgraduates preferred: a) pulpectomy and RCT for older patients in the presence or absence of symptoms; b) hydraulic calcium silicate cements as pulp capping material; and c) did not recommend systemic antibiotics.

**Clinical relevance:**

The majority of students choose non-selective (complete) caries removal in all cases and if the pulp is exposed, the use of hydraulic calcium silicate cements iwas the preferred material. Systemic antibiotics are considered unnecessary, irrespective of the patient's age and symptoms.

## Introduction

Historically, the management of deep caries involved the non-selective (complete) removal of all carious dentine.^1^ However, in recent times, a significant shift has occurred towards more minimally invasive biologically-based treatment strategies,^1^ including selective caries removal in one stage and two stages (stepwise excavation), as well as direct pulp capping, or partial and full pulpotomy. The aim of caries removal strategies in cases with no symptoms or symptoms not greater than those attributed to reversible pulpitis is to selectively remove caries in one or two visits, thereby minimising the risk of pulp exposure.^2^ Vital pulp treatments (VPT), which includes pulp capping, partial pulpotomy and full pulpotomy, have been advocated to treat teeth with pulp exposure, even in teeth that have signs and symptoms indicative of irreversible pulpitis.^3^, ^4^, ^5^ To guide the dental profession, the *European Society of Endodontology* (ESE)^1^ and the *American Association of Endodontists* (AAE)^6^ have published position statements that offer guidance, and support VPT as an endodontic treatment modality for the management of cariously-exposed pulps. However, the two statements differ in relation to some of their recommendations.

In order to reduce the risk of pulp exposure, the ESE indicates selective caries-removal strategies (one-stage selective carious-tissue removal to soft or firm dentin and two-stage stepwise excavation) in deep carious lesions of asymptomatic teeth or when the signs and symptoms indicate reversible pulpitis.^1^ However, the AAE advocates complete (nonselective) caries removal and the visualisation of the condition of the pulp under magnification in every case, implying that retaining soft dentin over the pulp compromises the observation of pulp inflammation levels and areas of potential pulp necrosis.^6^

Whilst VPT procedures have been advocated by prominent international organisations such as the ESE and AAE, previous surveys have shown that the majority of dental practitioners from France, Germany and the United Kingdom (UK) perform non-selective excavation of deep caries lesions, whereas most Norwegian dentists choose selective removal in two stages (stepwise excavation).^7^^,^^8^ The authors of these studies concluded that there was an imperative need to educate dentists about less invasive techniques. An international survey spanning 16 countries also reported that non-selective caries removal was preferred by dentists over selective caries removal.^9^ A survey of dental practitioners in Wales revealed that whilst high numbers of hospital-based practitioners undertook VPT procedures for managing cariously-exposed pulps, general dental practitioners reported a lack of training and material costs as barriers to adopting such minimally invasive endodontic treatment.^10^ A separate survey of the members of two European endodontic societies also investigated the management of pulp exposures, which revealed a lack of consensus regarding the ideal strategy for managing the exposed pulp and the preferred pulp capping material for VPT.^11^ In summary, various questionnaire-based studies from a range of countries have shown a lack of consensus on managing teeth with deep caries lesions and pulp exposures.^7^, ^8^, ^9^, ^10^, ^11^, ^12^, ^13^

The lack of consensus regarding both caries removal strategies and limited use of VPT amongst dental practitioners internationally may be related to the curriculum followed at dental schools. Given the advancements and recent recommendations made by global organisations^1^^,^^6^ in relation to minimally invasive techniques for managing deep carious lesions and exposed pulps, it is critical to evaluate dental students' knowledge of these techniques from a broader perspective as teaching philosophies regarding caries removal and VPT may differ across countries.

The primary objective of this survey was to evaluate the level of understanding regarding the treatment of deep carious lesions and exposed pulps among final-year dental undergraduate students and postgraduate endodontic students from ten dental institutions across ten countries. The secondary objective was to investigate the impact of operator (material, antibiotic prescription) and patient-related (age, symptoms) factors on the protocols used by students from the ten dental schools in relation to the treatment of teeth with deep carious lesions and exposed pulps.

## Methods

### Survey population

This survey included final-year undergraduate and postgraduate students (all years) in the field of endodontics at ten dental schools located in ten countries. In this survey, the term "undergraduate students" was employed to encompass all individuals pursuing a degree in general dentistry. Such students may already hold an undergraduate degree in another discipline and they are pursuing dentistry as graduate-entry students in some countries or universities.

### Ethical approval

The *a priori* protocol of this survey was obtained from the research ethics committee of the University of Sharjah (REC-22-11-29-01-F).

### Dental schools’ selection and ethical clearance

The project leader (VN) distributed an information sheet outlining the details of the project to one faculty member from each dental school via email to obtain their willingness to take part in the survey by distributing it to undergraduate and postgraduate students in their respective dental schools. Ten dental schools from different countries were selected based on geographical diversity and in order to obtain as broad a representation as possible. However, for consistency, if a dental school did not train postgraduate endodontic students, then that school was excluded from participation in the overall study. After obtaining the agreement of faculty members from participating schools, ethical clearance was obtained locally from each institution (Supplementary Table 1).

### Questionnaire

The Questionnaire was validated by six experts and piloted by 42 endodontic postgraduate students. The details of the validation and piloting process are provided in Supplementary Table 2. The finalised questionnaire had two sections (Supplementary Table 3):

*Section A*: General information about the participants.

Section B: This section included four case scenarios based on female patients presenting with deep caries in the mandibular first molar, with an intra-oral periapical radiograph and five multiple-choice questions for each case. The cases were of similar radiographic presentation (all inner ¼ of dentine), but differed in terms of the nature of the symptoms and the patient’s age.

### Survey process

After completion of the pilot study, the project leader (VN) finalised the online version of the questionnaire, which was subsequently distributed to the project co-leader (HD) and co-investigators (VY, NS) via email, Telegram and WhatsApp through a test link to verify the accurate display of the questions. Once confirmation was obtained, the project leader (VN) sent the details of the survey and requested the faculty members to disseminate it to all of their dental students who were eligible to take part in the study. The survey was conducted from March to May 2024. The students were duly informed that their involvement in the survey was not mandatory, that it would be anonymous, and that declining to participate or withdrawing from the survey would not affect their academic advancement or clinical progress. In an attempt to improve the response rate, several periodic reminders were sent to the student participants via email, Telegram, and WhatsApp, requesting that they complete the survey.

### Statistical analysis

The data were entered and analyzed statistically using SPSS software (Version 27.0, IBM Corp., USA). Simple descriptive statistics was used predominantly to describe the data. Additionally, McNemar's test was used to analyze paired categorical data, assessing significant changes in treatment choices between two related samples. A *P*-value of 0.05 was considered statistically significant, indicating a notable shift in preferences between scenarios that differed in terms of the tooth in question, the nature of the symptoms, and the patient’s age.

## Results

### Response rate

Table 1 presents the total number of students and responses obtained from the ten dental schools. The survey was completed by 435 undergraduates and 139 postgraduate students, out of a total of 1073 undergraduate and 152 postgraduate students who were eligible to participate, that is, 41% of undergraduates and 91% of postgraduates.

**Table 1:** The total number of responses received from the 10 dental schools

No.	Dental School, University, Country	Undergraduate students		Postgraduate students – Endodontics	
		Total number of students (n)	Total number of responses received n(%)	Total number of students (n)	Total number of responses received n(%)
1	UWA Dental School, The University of Western Australia, Australia	54	8 (14.81)	4	4 (100)
2	Faculty of Dentistry, University of British Columbia, Canada	59	26 (44.06)	9	8 (88.8)
3	Meenakshi Ammal Dental College and Hospital, Meenakshi Academy of Higher Education and Research, India	92	91 (98.91)	18	18 (100)
4	School of Dental Medicine, University of Belgrade, Serbia	176	68 (38.63)	33	33 (100)
5	Faculty of Dentistry, National University of Singapore, Singapore	73	23 (31.50)	11	11 (100)
6	School of Dentistry, Complutense University, Spain	74	35 (47.29)	18	17 (94.4)
7	Faculty of Dentistry, Yeditepe University, Turkey	100	92 (92.00)	17	16 (94.11)
8	College of Dentistry, Ajman University, United Arab Emirates	198	32 (16.16)	15	7 (46.66)
9	School of Dentistry, Cardiff University, United Kingdom	66	24 (36.36)	11	11 (100)
10	School of Dental Medicine, University of Pennsylvania, United States of America	181	36 (19.88)	16	14 (87.5)
	Total	1073	435 (40.54)	152	139 (91.44)

### Basic information

Table 2 provides the demographic information for undergraduate and postgraduate students within the ten schools. The mean ages of UGs and PGs were 24.3 and 30.3 years, respectively. There were 298 females and 137 male undergraduates who participated in the survey, while 86 females and 53 male postgraduates participated.

**Table 2:** Demographic data of the undergraduate and postgraduate students who participated in the survey

No.	Dental school, University, Country	Undergraduate (UG) / Post graduate (PG)	Age (years)		Gender		Number of teeth treated	
			Mean	Standard deviation	Female	Male	Mean	Standard deviation
1	UWA Dental School, The University of Western Australia, Australia	UG	25.38	3.02	5	3	7.13	17.33
		PG	35.00	6.06	1	3	22	22.35
2	Faculty of Dentistry, University of British Columbia, Canada	UG	29.92	4.48	15	11	5.65	5.99
		PG	32.75	4.86	4	4	12.5	17.93
3	Meenakshi Ammal Dental College and Hospital, Meenakshi Academy of Higher Education and Research, India	UG	21.63	0.96	68	23	1.77	1.61
		PG	26.11	1.32	11	7	20.35	27.88
4	School of Dental Medicine, University of Belgrade, Serbia	UG	25.04	1.49	49	19	2.09	2.58
		PG	33.30	5.27	21	12	26.23	41.06
5	Faculty of Dentistry, National University of Singapore, Singapore	UG	23.17	1.07	20	3	1.74	1.45
		PG	29.09	1.70	5	6	8.45	7.61
6	School of Dentistry, Complutense University, Spain	UG	23.77	2.25	31	4	2.87	3.01
		PG	28.76	4.93	11	6	28.12	25.27
7	Faculty of Dentistry, Yeditepe University, Turkey	UG	24.10	1.82	54	38	7.09	9.77
		PG	27.63	2.83	12	4	49.45	33.03
8	College of Dentistry, Ajman University, United Arab Emirates	UG	22.53	1.61	15	17	3.69	4.55
		PG	33.14	8.86	4	3	8.00	6.93
9	School of Dentistry, Cardiff University, United Kingdom	UG	22.83	0.70	14	10	1.88	1.62
		PG	29.64	2.46	7	4	3.00	6.05
10	School of Dental Medicine, University of Pennsylvania, United States of America	UG	30.09	4.84	27	9	2.17	2.38
		PG	30.64	3.03	10	4	51.57	41.57
	Over All	UG	24.31	3.41	298	137	3.54	6.01
		PG	30.28	4.94	86	53	24.37	32.64

Overall, 91% of both undergraduate students and postgraduate students were aware of the existence of guidelines or position statements specifically created for deep caries and exposed pulp. Seventy-six percent of the surveyed undergraduate students and 95% of the postgraduate students had treated teeth with deep caries lesions and an exposed pulp within the last 12 months. Eighty-six percent of the postgraduates were enrolled in a full-time course of which the majority were 3-year program.

### Responses provided by undergraduate and postgraduate students for the 4 case scenarios

The overall responses provided by undergraduate and postgraduate students for the four case-based scenarios are presented in Table 3. A small number of responses were excluded (Undergraduates: USA- 8 and Canada- 26; Postgraduates: USA- 4 and Canada- 8) from the analysis as a result of data errors or missing data discovered during the processing stage. Therefore, the final survey included 401 responses from undergraduates and 127 from postgraduates for analysis from nine dental schools.

**Table 3:** Overall response provided by undergraduates and postgraduate students for 4 case scenarios

Scenarios/Questions	Options	Undergraduates (%)	Postgraduates (%)
Scenario 1: A. How would you plan to treat tooth 46?	Non-selective (complete) caries removal	31.67	55.12
	Selective (partial) caries removal in one or 2 stages	68.33	44.88
Scenario 1: B. If pulp exposure occurred how would you plan to treat tooth 46?	Full coronal pulpotomy	6.98	4.72
	Partial Pulpotomy	17.71	28.35
	Pulp Capping	61.85	61.42
	Pulpectomy and root canal treatment	13.47	5.51
Scenario 1: C. If pulp capping or pulpotomy (partial or full) was selected as a treatment, what material would you choose?	Biodentine	37.16	67.72
	Hard setting calcium hydroxide	12.97	7.87
	Ledermix/Odontopaste	1.25	0
	Mineral trioxide aggregate	44.89	23.62
	Non-setting calcium hydroxide	3.49	0.79
	Others	0.25	0
Scenario 1: D. Provide justification for your answer choice in question C?	Expense	0.50	0
	I have been trained to use it at college/dental school	45.64	34.65
	I have read about it in the literature	41.90	43.31
	It is easy to handle	4.99	7.09
	It limits tooth discolouration	6.23	12.60
	Others	0.75	2.36
Scenario 1: E. Do you use systemic antibiotics while treating the tooth 46?	No	95	96.85
	Yes	5	3.15
Scenario 2: A. How would you plan to treat tooth 36?	Non-selective (complete) caries removal	57.86	80.31
	Selective (partial) caries removal in one or 2 stages	42.14	19.69
Scenario 2: B. If pulp exposure occurred how would you plan to treat tooth 36?	Full coronal pulpotomy	9.73	18.11
	Partial Pulpotomy	24.94	37.01
	Pulp Capping	29.93	18.11
	Pulpectomy and root canal treatment	35.41	26.77
Scenario 2: C. If pulp capping or pulpotomy (partial or full) was selected as a treatment, what material would you choose?	Biodentine	33.67	59.84
	Hard setting calcium hydroxide	14.46	3.15
	Ledermix/Odontopaste	2.99	2.36
	Mineral trioxide aggregate	37.91	28.35
	Non-setting calcium hydroxide	6.48	1.57
	Others	4.49	4.72
Scenario 2: D. Provide justification for your answer choice in question C?	Expense	0.50	0.79
	I have been trained to use it at college/dental school	41.65	36.22
	I have read about it in the literature	44.14	41.73
	It is easy to handle	5.49	7.87
	It limits tooth discolouration	5.99	7.87
	Others	2.24	5.51
Scenario 2: E. Do you use systemic antibiotics while treating the tooth 46?	No	96	100.00
	Yes	4	0
Scenario 3: A. How would you plan to treat tooth 36?	Nonselective (complete) caries removal	51.87	76.38
	Selective (partial) caries removal in one or 2 stages	48.13	23.62
Scenario 3: B. If pulp exposure occurred how would you plan to treat tooth 36?	Full coronal pulpotomy	15.71	19.69
	Partial Pulpotomy	19.45	20.47
	Pulp Capping	23.19	27.56
	Pulpectomy and root canal treatment	41.65	32.28
Scenario 3: C. If pulp capping or pulpotomy (partial or full) was selected as a treatment, what material would you choose?	Biodentine	33.17	60.63
	Hard setting calcium hydroxide	17.46	4.72
	Ledermix/Odontopaste	3.74	0.79
	Mineral trioxide aggregate	36.16	25.20
	Nonsetting calcium hydroxide	5.74	1.57
	Others	3.74	7.09
Scenario 3: D. Provide justification for your answer choice in question C?	Expenses	1.25	0
	I have been trained to use it at college/dental school	41.40	34.65
	I have read about it in the literature	42.39	46.46
	It is easy to handle	7.98	8.66
	It limits tooth discolouration	5.74	5.51
	Others	1.25	4.72
Scenario 3: E. Do you use systemic antibiotics while treating the tooth 36?	No	98	94.49
	Yes	2	5.51
Scenario 4: A. How would you plan to treat tooth 46?	Nonselective (complete) caries removal	53.37	79.53
	Selective (partial) caries removal in one or 2 stages	46.63	20.47
Scenario 4: B. If pulp exposure occurred how would you plan to treat tooth 46?	Full coronal pulpotomy	11.72	20.47
	Partial pulpotomy	17.96	22.05
	Pulp Capping	32.17	15.75
	Pulpectomy and root canal treatment	38.15	41.73
Scenario 4: C. If pulp capping or pulpotomy (partial or full) was selected as a treatment, what material would you choose?	Biodentine	35.41	52.76
	Hard setting calcium hydroxide	13.47	5.51
	Ledermix/Odontopaste	3.74	2.36
	Mineral trioxide aggregate	36.16	26.77
	Nonsetting calcium hydroxide	6.73	2.36
	Others	4.49	10.24
Scenario 4: D. Provide justification for your answer choice in question C?	Expense	2.00	0.79
	I have been trained to use it at college/dental school	41.90	31.50
	I have read about it in the literature	41.15	51.18
	It is easy to handle	7.98	3.94
	It limits tooth discolouration	5.49	4.72
	Others	1.50	7.87
Scenario 4: E. Do you use systemic antibiotics while treating the tooth 46?	No	96.3	95.28
	Yes	3.7	4.72

*Analysis includes 401 responses from undergraduates and 127 from postgraduates.

### Selective vs nonselective caries removal

The presence or absence of symptoms significantly altered treatment planning decisions in young patients (case scenarios 1 and 2; *P* < .001) for both undergraduate and postgraduate students. In young patients, if symptoms were present (case scenario 2), most undergraduate and postgraduate students preferred a non-selective (complete) caries removal technique compared with selective (partial) caries removal (Table 3). Conversely, in the absence of symptoms (case scenario 1), the majority of undergraduates preferred selective (partial) caries removal, whereas postgraduates still preferred non-selective (complete) caries removal (Table 3).

The presence or absence of symptoms makes no significant change to the treatment planning decisions for older patients (case scenarios 3 and 4) by both undergraduate (*P* = .709) and postgraduate (*P* = .541) students. In older patients, (case scenarios 3 and 4), if symptoms are present or absent, the majority of undergraduate and postgraduate students preferred a non-selective (complete) caries removal approach.

For both undergraduate and postgraduate students, there was a significant difference between treatment plans for younger (Case 1) and older patients (Case 3) in the absence of symptoms (*P* < .001), with more respondents electing to remove all the caries in older patients compared with younger patients. However, there was no difference between younger (Case 2) and older patients (Case 4) if symptoms were present (undergraduates, *P* = .12; postgraduates, *P* = 1.000)).

### Pulp capping vs partial pulpotomy vs full coronal pulpotomy vs pulpectomy and root canal treatment

The presence or absence of symptoms significantly altered treatment planning decisions in young patients (case scenarios 1 and 2) by both undergraduate (*P* < .001) and postgraduate (*P* < .001) students with more invasive treatments preferred. Pulp capping was preferred less when symptoms were present and there was a tendency to perform more pulpectomy and RCT when symptoms were present in younger patients (Table 3). However, the presence or absence of symptoms had no significant effect on the treatment planning decisions for older patients (case scenarios 3 and 4) by both undergraduate (*P* = .051) and postgraduate students (*P* = .273).

If symptoms were present, most of the undergraduate students preferred pulpectomy and RCT for both young and old patients, whereas most of the postgraduates preferred partial pulpotomy for young patients and pulpectomy and RCT for older patients with spontaneous pain.

For both undergraduate and postgraduate students, there was a significant difference between younger and older patients’ treatment plans in the presence or absence of symptoms with more invasive treatments preferred for older patients.

### Material of choice

The majority of undergraduates selected MTA, followed by Biodentine, as their preferred material of choice, whereas the majority of postgraduates selected Biodentine, followed by MTA, as their preferred material for pulp capping or pulpotomy (partial or full) procedures (Table 3). For both undergraduates and postgraduates, the most common reasons for selecting the materials were that they had been "trained to use them at college" and they "have read about them in the literature."

### Use of antibiotics

More than 94% of undergraduates and postgraduates did not recommend the use of systemic antibiotics when treating any of the outlined cases. Among both undergraduate and postgraduate students, the patient's age and symptoms did not alter the prescription of systemic antibiotics.

## Discussion

This is the first survey to assess the knowledge and behaviour of undergraduate and postgraduate students from ten dental schools across ten countries regarding the management of deep caries and exposed pulp. The results of this study will contribute to the assessment of students' comprehension in this field, facilitating the development of existing curricula in this area and improving the standardisation of treatment protocols for the benefit of patients.

The current survey received a lower response rate from undergraduate students (41%) compared with their postgraduate counterparts (91%). A greater awareness of current research in this domain amongst postgraduate students with increased subject-driven motivation and interest could potentially explain the disparity in response rates between undergraduate and postgraduate students.^14^ A recent survey, which aimed to assess the knowledge of undergraduate and postgraduate students from ten dental schools across ten countries about the treatment of traumatic dental injuries, identified a similar pattern in terms of responses.^14^

Therapeutic strategies that non-selectively remove caries in teeth with deep caries and no or mild symptoms have been labeled as over-treatment and outdated in position statements from cariology groups.^15^ Furthermore, the ESE supported this by recommending the avoidance of pulp exposure using selective caries removal in one or two stages, when the signs and symptoms were indicative of no more than reversible pulpitis.^1^ This recommendation was based on 5-year randomised controlled trial data for teeth with deep caries that highlighted superior results for selective removal techniques. ^16^^,^^17^ Notably, the AAE suggested non-selective removal of caries in every deep caries case,^6^ a recommendation that led to calls for consensus and union between leading organisations for dentist and patient benefit.^18^ When caries removal techniques are closely compared, the success rate of non-selective caries removal after 5 years was shown to be 46.3% in comparison with stepwise caries removal at 60.2%; however, that paper considered pulp exposure as a failure which may be misleading.^17^ Avoiding exposure of the pulp by using less invasive strategies such as a single-stage (partial) or 2-step caries excavation in asymptomatic teeth with signs and symptoms indicative of reversible pulpitis has been recommended.^1^ In a recent meta-analysis, among the less invasive caries excavation methods, single-stage caries removal that avoids later re-entry, was considered superior to 2-stage stepwise excavation in terms of clinical outcome at 1.5 years. However, after 5 years, the treatment outcome with both techniques was similar.^19^ Another systematic review argued in favour of one-stage partial caries excavation over stepwise excavation, stating the latter needs multiple visits and risks pulp exposure during re-entry. In the systematic review, the success rate of partial caries excavation was 1.12 times greater than stepwise, a finding that was attributed to low patient compliance at the second visit.^20^ Notably, the ESE position statement^1^ did not recommend selective caries removal if more severe symptoms, including spontaneous pain, were present because there is no evidence to support it given that the only studies available were case reports on immature teeth.^21^ This message seemed to have resonated with the respondents in the current study as symptoms significantly changed treatment plans towards non-selective caries removal approaches.

A retrospective study of different vital pulp treatment options after carious pulp exposures has shown that there is no difference between the survival rates of full pulpotomy, partial pulpotomy and direct pulp capping. However, the outcome of VPT was associated with factors such as pulp and periapical status, restoration type and restoration surfaces.^22^ Although full pulpotomy has been reported to have a high success rate after three years in a recent systematic review,^23^ pulp capping after carious exposure has recently been reported to have particularly inferior results after four years,^24^ potentially due to the lack of removal of superficially inflamed or infected pulp tissues. A meta-analysis revealed higher success rates for direct pulp capping with MTA or Biodentine (84% and 86%) in teeth with carious pulp exposures compared with that of calcium hydroxide (59%) over three years follow up.^25^ Interestingly, until recently no studies were available that have compared indirect or direct pulp capping with pulpotomy which probably could be attributed to the ethical issues in designing a trial with one arm having non-selective caries removal leading to the risk of a higher rate of pulp exposures.^26^ In 2023, one randomised trial circumvented the ethical issues and investigated selective caries removal versus pulpotomy for the management of deep caries.^27^ Although they noted a 91.4% survival rate at 1-year, selective caries removal led to more pulpectomies than pulpotomy.^27^ According to a clinical trial conducted with a 2-year follow-up, the success rate of selective caries removal to soft dentine was higher than that of selective removal to firm dentine in the treatment of deep carious lesions^28^. In another clinical trial, it was reported that both selective removal to soft dentine and full pulpotomy had high success rates when used to treat permanent mandibular molar teeth with deep carious lesions.^29^ However, pulpotomy has proven to be an alternate therapy for RCT in cases of cariously-exposed pulps with an overall success rate of 85% over 18 months.^30^ A recent meta-analysis showed pooled clinical and radiographic success rates for pulpotomy were 92.9% and 78.5%, respectively, and it was concluded that pulpotomy was a viable alternative to pulpectomy in cases with signs and symptoms indicative of irreversible pulpitis.^31^ Within the current study, symptoms did change the choice of procedure with respondents electing to remove more pulp tissue when there were symptoms, a finding that was more pronounced when the patients were older. This trend is logical as studies investigating VPT for teeth with signs and symptoms indicative of irreversible pulpitis have invariably not used a pulp capping technique,^32^ while classic teaching has prescribed RCT for pulp exposures with signs and symptoms indicative of irreversible pulpitis.^33^ These results are in line with Tomson et al. who stated that pulpotomy is a treatment modality that is as effective as RCT. The associated meta-analysis revealed no difference in post-operative pain (Day 7) between RCT and pulpotomy with high clinical success at year 1 (98%) for both interventions.^34^

In the current survey, the majority of undergraduates preferred pulpectomy and RCT for both young and older adults when symptoms were present. Conversely, the majority of postgraduates preferred partial pulpotomy for young patients and pulpectomy and RCT for older patients. There are several possible explanations for these trends, including the possibility that undergraduates possess a lower level of understanding of VPT and potentially a lower level of confidence in performing VPT compared with postgraduates. Alternatively, it may highlight that recent evidence of VPT techniques has not yet filtered down to dental curricula yet.

The treatment regimens of younger and older patients differed significantly for both undergraduate and postgraduate students. Similarly, a survey conducted by the Irish Endodontic Society and the Accademia Italiana di Endodonzia indicated that the decision-making process was significantly influenced by the age of the patients.^11^ This is consistent with another survey among Swedish dentists that studied operator choice in a variety of scenarios and identified that more invasive options were generally selected for older patients.^35^ Notably, however, age has not been shown to a reliable predictor of outcomes for VPT with similar levels of successes and failures irrespective of patient age.^36^, ^37^, ^38^

In the current survey, the majority of undergraduate and postgraduate students stated that they would use a hydraulic calcium silicate cement (such as MTA or Biodentine). The most common reasons for selecting these materials appeared to be that they had been ‘trained to use them at college’ and ‘students have read about them in the literature’. This shows that histological^39^ and clinical evidence^40^ has highlighted the benefits of hydraulic calcium silicate cements compared with other materials and that dental schools have integrated the teaching of hydraulic calcium silicate cements into their undergraduate and postgraduate curricula. The ESE position statement^1^ recommends the use of hydraulic calcium silicate cements for managing teeth with deep caries and pulp exposures. In a study comparing members of two European endodontic societies, hydraulic calcium silicate cements, such as Biodentine and MTA, were frequently chosen as VPT materials, being the material of choice for 69% of Accademia Italiana di Endodonzia (AIE) members and 81% of Irish Endodontic Society members who responded to the survey. However, oddly, younger members of the AIE preferred calcium hydroxide.^11^ A recent survey among Greek dentists reported that for pulpotomy, the most frequently used material was MTA, followed by other bioceramics and calcium hydroxide.^41^ In a recent systematic review, Biodentine compared positively with MTA in terms of overall clinical success as a DPC material.^25^

In the current survey, more than 95% of the undergraduates and postgraduates did not recommend the use of systemic antibiotics and interestingly the patient's age and symptoms did not alter this recommendation. Similar to these results, nearly all members of two European endodontic societies indicated that antibiotic use for the treatment of profound carious lesions and exposed pulps was unnecessary.^11^ The evidence suggests that antibiotics prescribed for pulp-related pain provide minimum/negligible benefits and probably contribute to harmful effects such as the development of antibiotic resistance.^42^

This study included students from 10 different countries, which could be considered a strength of the study. However, the study was subject to the following limitations: (1) During the survey, there may have been variations in students' knowledge levels across different countries. For example, the academic year commences in September in the United Arab Emirates and in January in Australia; (2) the principal investigators (VN, HD) selected dental schools primarily through personal contacts in the ten countries, which although practical, highlights a possible selection bias. A more systematic selection process in the selection of participating countries could improve geographic representation; (3) one dental school from each country was chosen and therefore, it is not realistic to claim that it precisely represents students throughout each country and it may lack generalisability in some instances; (4) some responses were excluded from the analysis due to data errors or incompleteness. During this process due to a misunderstanding, all the responses from one dental school in Canada were excluded. Repeating the entire survey, or that particular question, may have caused bias and compromised the survey's validity; (5) the level of knowledge of students from the different schools has not been compared in the current survey as there was a variation in the response rates from the ten schools. The power and validity of inferential statistics might be influenced by this variability; (6) The confidentiality of each participating institution prevented the disclosure of their curriculum details; (7) the current survey did not attempt to gather all the details regarding the procedure for managing deep caries and exposed pulps, such as the size of the exposure and the concentration of hemostatic solution placed on the pulp exposure site. Therefore, it is imperative that a future survey be conducted to perform an in-depth evaluation of the treatment protocols; (8) The limitation of any questionnaire survey is having response bias such as providing socially desirable replies not reflecting the true opinion of the participant as well as enhancing knowledge with AI tools or books; and (9) The current survey included postgraduates only from the endodontic discipline. Future surveys should involve postgraduates from other disciplines to ensure a comprehensive approach.

To improve knowledge, confidence and standardisation among students, the authors of the current survey propose the following recommendations:

1. The curricula of any dental school should be in accordance with the evidence-based guidelines that have been developed within the respective country.
2. It is imperative that each dental school implement an exercise to establish consistency among faculty members within their respective fields and across other disciplines, which in turn ensures that the delivery is consistent.
3. The curriculum must allocate more time to didactic lectures, preclinical and clinical training in deep caries and exposed pulp.
4. The knowledge and confidence of students would be enhanced in this area by the inclusion of new online courses and educational videos in the curriculum.
5. Faculty members should encourage students to attend national and international conferences, and symposia to update their knowledge. This can also be part of the curriculum.

## Conclusions

• The majority of undergraduates and postgraduates preferred non-selective (complete) caries removal even in the absence of symptoms.
• Most undergraduates and postgraduates would choose pulp capping as the treatment of choice for a young patient with no symptoms. However, symptoms led to undergraduate students preferring pulpectomy and RCT, while postgraduates preferred partial pulpotomy. The most commonly selected option for both undergraduates and postgraduates was pulpectomy and RCT for older patient’s for both the presence or absence of symptoms.
• Hydraulic calcium silicate cements are the preferred choice of the majority of undergraduates and postgraduates.
• The use of systemic antibiotics was not recommended by the majority of undergraduates and postgraduates.
• Within the limitations of this survey, VPT was a popular treatment option particularly for young patients. However, there was considerable disparity in decision-making between undergraduate and postgraduate students.

## Author contributions

Conceptualisation: V.N, P.A, H.D; Methodology: V.N, A.D, A.A, A.J, A.H, F.S, J.I, M.B, M.K, N.S, P.A, R.S, S.N, V.Y, V.G, Y.S, H.D; Data Curation: V.N, P.A, H.D, S.N, V.G; Formal analysis: V.N, P.A, H.D, S.N, V.G;; Project administration: V.N, H.D; Supervision: V.N, H.D, P.A; Manuscript writing: V.N, A.D, A.A, A.J, A.H, F.S, J.I, M.B, M.K, N.S, P.A, R.S, S.N, V.Y, V.G, Y.S, H.D;; Manuscript revision and approval: V.N, A.D, A.A, A.J, A.H, F.S, J.I, M.B, M.K, N.S, P.A, R.S, S.N, V.Y, V.G, Y.S, H.D.

## Ethical statement

The ethical clearance was approved by the following institutional ethical committees: 1. Ajman University (D-F-H-10-Jan-A), United Arab Emirates; 2. Cardiff University (2302), United Kingdom (UK); 3. Complutense University (CE_20221215-16_SAL), Spain; 4. University of Belgrade (No 36/3), Serbia; 5. University of British Columbia (H24-00123), Canada; 6. University of Pennsylvania (853452), United States of America (USA); 7. Yeditepe University (E.83321821-805.02.03-129), Turkey; 8. Meenakshi Academy of Higher Education and Research (MADC/IEC-I/1/2023), India; 9. University of Western Australia (2022/ET001037), Australia; 10. National University of Singapore (NUS-IRB-2023-52), Singapore, 11. University of Sharjah (REC-22-11-29-01-F), UAE.

## Conflict of interest

None disclosed.

## Appendix Supplementary materials

Image, application 1

## Footnotes

Supplementary material associated with this article can be found in the online version at doi:10.1016/j.identj.2025.100844.
