# Supplementary material for: A Multi-National Questionnaire-Based Analysis of Dental Students’ Knowledge of the Management of Deep Caries and the Exposed Pulp
Source: Int Dent J. 2025 Jun 6;75(4):100844. doi: 10.1016/j.identj.2025.100844 (PMC12173050; doi:10.1016/j.identj.2025.100844)
Supplement: Supplementary file 1 [file mmc1.docx]

**Supplementary Table 1: Details of ethical clearance**

| 1. University of Pennsylvania (853452), United States of America (USA);  2. University of British Columbia (H24-00123), Canada;  3. University of Belgrade (No 36/3), Serbia;  4. Meenakshi Academy of Higher Education and Research (MADC/IEC-I/1/2023), India;  5. Yeditepe University (E.83321821-805.02.03-129), Turkey;  6. Cardiff University (2302), United Kingdom (UK);  7. National University of Singapore (NUS-IRB-2023-52), Singapore;  8. Complutense University (CE_20221215-16_SAL), Spain;  9. Ajman University (D-F-H-10-Jan-A), United Arab Emirates (UAE);  10. University of Western Australia (2022/ET001037), Australia) |
| --- |

**Supplementary Table 2: Details of the validation and piloting process**

| *Validation of the questionnaire*  The questionnaire used in this survey was developed by the project leaders (VN, HD) through the adaptation of a previously published questionnaire [1]. The current questionnaire was validated by a panel of six experts with more than ten years of academic and/or clinical experience in endodontics. The experts were given a scale from 1 to 4 to assess the items based on relevance, clarity, simplicity, and ambiguity in line with accepted methodology [2. In the present survey, two content validity indices (CVI) were calculated: the content validity of each individual item (I-CVI), and the average content validity of the entire scale (S-CVI). The individual item's content validity was determined by dividing the total number of experts' who have rated 3 or 4 (experts in agreement) by the total number of experts. The S-CVI was computed by averaging the I-CVI. The minimum value for S-CVI should be 0.83 [3]. Additionally, experts were given the opportunity to provide feedback regarding the order of items within the questionnaire, its grammatical structure, and the need for any addition or removal of items. The validation process ended upon the completion of the second round. All items in the survey received a mean I-CVI of 0.83 to 1 for relevance, clarity, simplicity, and ambiguity. S-CVI was 0.88, 0.93, 0.93 and 0.89 for relevance, clarity, simplicity, and ambiguity, respectively. Based on the above I-CVI and S-CVI scores, it was agreed that the scale of the questionnaire achieved a satisfactory level of content validity.  *Piloting the questionnaire*  After completing the validation process, the online version of the questionnaire was prepared using Google Docs/ Qualtrics (Qualtrics, Seattle, WA, USA)/ Online Surveys (Jisc, Bristol, UK).  In total, 42 endodontic postgraduate students from India and the UAE participated in the pilot study and these students were excluded from the final survey. The postgraduate students scored all the items in the survey and were also given the opportunity to provide feedback pertaining to the flow of items in the questionnaire, the level of simplicity in understanding the questions, and the time required to complete the survey. Analysis of the results showed that 93% and 100% of the students reported the flow of the questions to be logical, and easy to understand, respectively. The majority of the students (88%) completed the survey within seven minutes.  **References**   1. Careddu R, Plotino G, Cotti E, Duncan HF (2021) The management of deep carious lesions and the exposed pulp amongst members of two European endodontic societies: a questionnaire‐based study. Int Endod J 54: 366-376. https://doi:10.1111/iej.13418. 2. Abdulkader Mohamed R, Abdul Rahim NA, Mohamad SM, Ahmad Yusof H (2022) Validity and reliability of knowledge, attitude, and practice regarding exercise and exergames experiences questionnaire among high school students. BMC Public Health 22: 1743. 3. Yusoff MSB (2019) ABC of content validation and content validity index calculation. Education in Medicine Journal 11: 49–54. |
| --- |

**Supplementary Table 3: Questionnaire**

| **Questionnaire for Undergraduate students**  **SECTION A**   1. What is your gender? 2. Male 3. Female 4. What is your age? 5. Name of your University? 6. Name of the Country, your university is located in? 7. Have you ever treated teeth with deep caries lesions and exposed pulp, if so, approximately how many teeth in the last 12 months? 8. Are you aware of existence of guidelines or position statements for management of deep caries and the exposed pulp? 9. Yes 10. No   **Questionnaire for Postgraduate students**  **SECTION A**   1. What is your gender? 2. Male 3. Female 4. What is your age? 5. Name of your University? 6. Name of the Country, your university is located in? 7. What is the length of your postgraduate course or speciality training (i.e. how many years)? 8. Currently, in which year are you enrolled in a postgraduate program or specialized training 9. Is the course full-time? 10. Have you ever treated teeth with deep caries lesions and exposed pulp, if so, approximately how many teeth in the last 12 months? 11. Are you aware of existence of guidelines or position statements for management of deep caries and the exposed pulp? 12. Yes 13. No   **SECTION - B**   \| **Scenario 1** \| \| \| --- \| --- \| \| **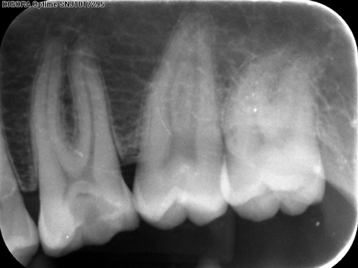** \| An 18-year-old female patient with a non-contributory  medical history presents with a deep carious lesion  (inner ¼ of dentine on radiograph) on 46 with no  symptoms or spontaneous nocturnal pain. Pulp sensibility  tests elicit a positive response. Please provide your  answers for the following questions relating to this scenario. \| \| 1. How would you plan to treat tooth 46?  - Selective (partial) caries removal in one or two stages - Non-selective (complete) caries removal \| \| \| 1. If pulp exposure occurred how would you plan to treat tooth 46?  - Pulp Capping - Partial Pulpotomy - Full coronal pulpotomy - Pulpectomy and root canal treatment \| \| \| 1. If pulp capping or pulpotomy (partial or full) was selected as a treatment, what material would you choose?  - Mineral trioxide aggregate - Biodentine - Hard setting calcium hydroxide - Non-setting calcium hydroxide - Ledermix/Odontopaste - Others ………. \| \| \| 1. Provide justification for your answer choice in question C?  - I have read about it in the literature - I have been trained to use it at college/dental school - It limits tooth discolouration - It is easy to handle - Expense - Other ------- \| \| \| 1. Would you consider using systemic antibiotics while treating the tooth 46?  - Yes - No \| \|  \| **Scenario 2** \| \| \| --- \| --- \| \| **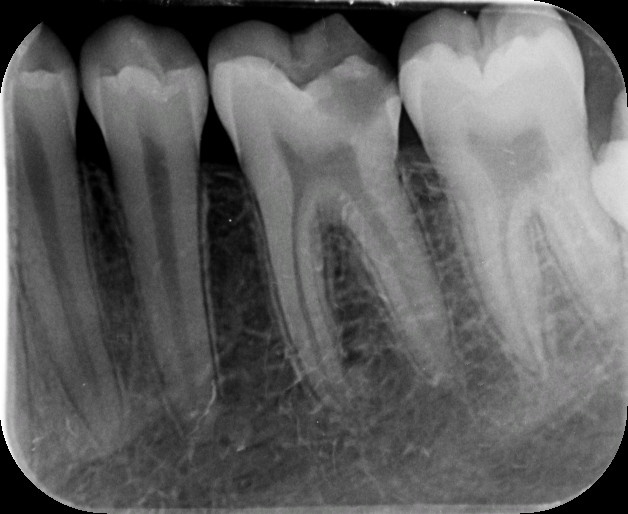** \| An 18-year-old female patient with a  non-contributory medical history  presenting with a deep carious lesion  (inner ¼ of dentine on radiograph) on  36 associated with moderate  symptoms triggered by cold/sweet and  tenderness when biting as well as  spontaneous pain. Pulp sensibility test  elicit a positive response. Please provide  your answers for the following questions relating to this scenario. \| \| 1. How would you plan to treat tooth 36?  - Selective (partial) caries removal in one or two stages - Non-selective (complete) caries removal \| \| \| 1. If pulp exposure occurred how would you plan to treat tooth 36?  - Pulp Capping - Partial Pulpotomy - Full coronal pulpotomy - Pulpectomy and root canal treatment \| \| \| 1. If pulp capping or pulpotomy (partial or full) was selected as a treatment, what material would you choose?  - Mineral trioxide aggregate - Biodentine - Hard setting calcium hydroxide - Non-setting calcium hydroxide - Ledermix/Odontopaste - Others ………. \| \| \| 1. Provide justification for your answer choice in question C?  - I have read about it in the literature - I was trained to use it at college - It limits tooth discolouration - It is easiest to handle - Expense - Other ------- \| \| \| 1. Do you use systemic antibiotics while treating the tooth 36?  - Yes - No \| \|  \| **Scenario 3** \| \| \| --- \| --- \| \| 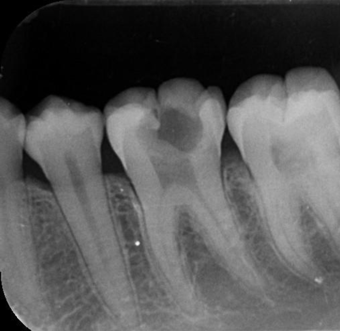 \| A 50-year-old female patient with a non-contributory medical  history presents with deep carious lesion (inner ¼ of  dentine on radiograph) on 36 with no symptoms or  spontaneous nocturnal pain. Pulp sensibility tests elicit a  positive response. Please provide your answers for the  following questions relating to this scenario. \| \| 1. How would you plan to treat tooth 36?  - Selective (partial) caries removal in one or two stages - Non-selective (complete) caries removal \| \| \| 1. If pulp exposure occurred how would you plan to treat tooth 36?  - Pulp Capping - Partial Pulpotomy - Full coronal pulpotomy - Pulpectomy and root canal treatment \| \| \| 1. If pulp capping or pulpotomy (partial or full) was selected as a treatment, what material would you choose?  - Mineral trioxide aggregate - Biodentine - Hard setting calcium hydroxide - Non-setting calcium hydroxide - Ledermix/Odontopaste - Others ………. \| \| \| 1. Provide justification for your answer choice in question C?  - I have read about it in the literature - I was trained to use it at college - It limits tooth discolouration - It is easiest to handle - Expense - Other ------- \| \| \| E. Do you use systemic antibiotics while treating the tooth 36?   - Yes - No \| \|  \| **Scenario 4** \| \| \| --- \| --- \| \| **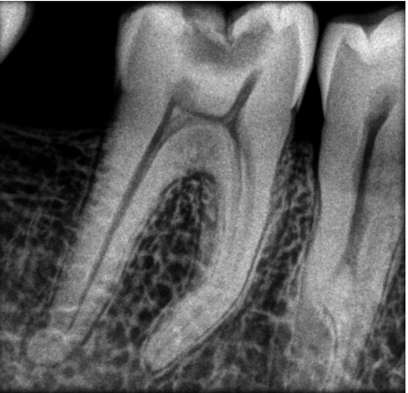** \| A 50-year-old female patient with a non-contributory medical  history presents with deep carious lesion (inner ¼ of dentine  on radiograph) on 46 associated with moderate symptoms  triggered by cold/sweet and tenderness when biting as well  as spontaneous pain. Pulp sensibility tests elicit a positive  response. Please provide your answers for the following  questions relating to this scenario. \| \| 1. How would you plan to treat tooth 46?  - Selective (partial) caries removal in one or two stages - Non-selective (complete) caries removal \| \| \| 1. If pulp exposure occurred how would you plan to treat tooth 46?  - Pulp Capping - Partial Pulpotomy - Full coronal pulpotomy - Pulpectomy and root canal treatment \| \| \| 1. If pulp capping or pulpotomy (partial or full) was selected as a treatment, what material would you choose?  - Mineral trioxide aggregate - Biodentine - Hard setting calcium hydroxide - Non-setting calcium hydroxide - Ledermix/Odontopaste - Others ………. \| \| \| 1. Provide justification for your answer choice in question C?  - I have read about it in the literature - I was trained to use it at college - It limits tooth discolouration - It is easiest to handle - Expense - Other ------- \| \| \| E. Do you use systemic antibiotics while treating the tooth 46?   - Yes - No \| \| |
| --- | --- | --- | --- | --- | --- | --- | --- | --- | --- | --- | --- | --- | --- | --- | --- | --- | --- | --- | --- | --- | --- | --- | --- | --- | --- | --- | --- | --- | --- | --- | --- | --- | --- | --- | --- | --- | --- | --- | --- | --- | --- | --- | --- | --- | --- | --- | --- | --- | --- | --- | --- | --- | --- | --- | --- | --- |
